# Effectiveness of EGFR tyrosine kinase inhibitors in advanced non‐small cell lung cancer patients with uncommon *EGFR* mutations: A multicenter observational study

**DOI:** 10.1111/1759-7714.13718

**Published:** 2020-10-29

**Authors:** Masaki Kanazu, Masahide Mori, Madoka Kimura, Kazumi Nishino, Takayuki Shiroyama, Izumi Nagatomo, Shoichi Ihara, Kiyoshi Komuta, Hidekazu Suzuki, Tomonori Hirashima, Toru Kumagai, Fumio Imamura

**Affiliations:** ^1^ Department of Thoracic Oncology National Hospital Organization Osaka Toneyama Medical Center Toyonaka Japan; ^2^ Department of Thoracic Oncology Osaka International Cancer Institute Osaka Japan; ^3^ Department of Respiratory Medicine and Clinical Immunology Osaka University Graduate School of Medicine Suita Japan; ^4^ Department of Respiratory Medicine Osaka Police Hospital Osaka Japan; ^5^ Department of Respiratory Medicine Daini Osaka Police Hospital Osaka Japan; ^6^ Department of Thoracic Oncology Osaka Prefectural Hospital Organization Osaka Habikino Medical Center Habikino Japan

**Keywords:** Afatinib, epidermal growth factor receptor, erlotinib, gefitinib, uncommon mutation

## Abstract

**Background:**

Epidermal growth factor receptor‐tyrosine kinase inhibitor (EGFR‐TKI) therapy is the standard treatment for advanced non‐small cell lung cancer (NSCLC) harboring common *EGFR* mutations, such as exon 19 deletion or L858 point mutation. However, the effectiveness of EGFR‐TKIs for patients with uncommon *EGFR* mutations remains unclear.

**Methods:**

We retrospectively surveyed a consecutive database of NSCLC patients with *EGFR* mutations at five participating institutions. Data from NSCLC patients with uncommon mutations (including single or compound mutations), who were treated with systemic therapy between May 2016 and October 2018, were collected and analyzed.

**Results:**

A total of 23 of the 524 patients whose data were collected had uncommon *EGFR* mutations. Of these, 22 received EGFR‐TKIs (gefitinib = 6, erlotinib = 4, and afatinib = 12). Patients who received first EGFR‐TKIs had overall response and disease control rates of 59.1% and 81.8%, respectively. The median progression‐free survival (PFS) of patients with G719X mutation (*n* = 13, median PFS = 32.9 months) was favorable, compared with those of patients with L861Q mutation (*n* = 4, median PFS = 6.4 months) and compound mutations (*n* = 4, median PFS = 7.3 months). The PFS of patients who received first‐ and second‐generation EGFR‐TKIs was 14.0 months (*n* = 10) and 7.3 months (*n* = 12), respectively. The median cumulative duration of treatment (DOT) with EGFR‐TKIs was 30.4 months, which was longer than those of cytotoxic chemotherapy (median DOT = 10.7 months) or immune checkpoint inhibitors (median DOT = 6.6 months).

**Conclusions:**

EGFR‐TKIs elicit favorable responses and contribute to long‐term disease control in NSCLC patients with uncommon *EGFR* mutations.

**Key points:**

**Significant findings of the study**: Our results demonstrate that both first‐ and second‐generation EGFR‐TKIs elicit favorable responses in NSCLC patients with uncommon *EGFR* mutations.

**What this study adds** This study revealed all clinical courses for NSCLC patients with uncommon *EGFR* mutations. In addition to EGFR‐TKIs, CCT and ICIs were found to contribute to long‐term disease control in this cohort.

## Graphical abstract

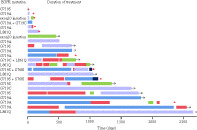

This study revealed all clinical courses for NSCLC patients with uncommon *EGFR* mutations. In addition to EGFR‐TKIs, cytotoxic chemotherapy (CCT) and immune checkpoint inhibitors (ICIs) contributed to long‐term disease control in this cohort.

## Introduction

Epidermal growth factor receptor‐tyrosine kinase inhibitors (EGFR‐TKIs) have been reported to induce a dramatic response in tumors harboring activating *EGFR* mutations. Two major types of *EGFR* mutations, in‐frame deletional mutation in exon 19 and L858R point mutation in exon 21, have been confirmed to confer sensitivity to EGFR‐TKIs.^1^, ^2^, ^3^, ^4^, ^5^ Other mutations, such as amino acid substitutions in G719, S768, and L861, constitute approximately 10% of all *EGFR* mutations.^6^, ^7^, ^8^, ^9^ Although preclinical models demonstrate that uncommon mutations respond to EGFR‐TKIs to some extent,^10^, ^11^ the clinical effectiveness of EGFR‐TKIs in non‐small cell lung cancer (NSCLC) patients with these uncommon mutations remains unclear.

Retrospective studies on first‐generation EGFR‐TKIs gefitinib and erlotinib have shown variable responses in NSCLC patients with uncommon mutations.^12^, ^13^, ^14^, ^15^ In contrast, second‐generation EGFR‐TKI afatinib has been reported to elicit a favorable response in patients with uncommon *EGFR* mutations, according to the LUX‐Lung clinical trials (a single group phase 2 trial [LUX‐Lung 2] and randomized phase 3 trials [LUX‐Lung 3 and 6]).^16^ However, the effectiveness of EGFR‐TKIs in clinical practice for NSCLC patients harboring uncommon *EGFR* mutations remain controversial because of the lack of cohesive reports.

In this study, we investigated the clinical features of NSCLC patients with uncommon *EGFR* mutations (including single or compound mutations) treated with first‐ and second‐generation EGFR‐TKIs. Furthermore, we focused on all clinical courses for this cohort, including cytotoxic chemotherapy (CCT) and immune checkpoint inhibitors (ICIs).

## Methods

### Patients and data collection

We performed a retrospective survey of a consecutive database of locally advanced or metastatic NSCLC (pathologically or cytologically proven) patients with *EGFR* mutations at five participating institutions. Patients with NSCLC harboring *EGFR* mutations were enrolled in the study if: (i) they were on any systemic therapy on 25 May 2016 and anticipated a new therapy after disease progression (PD); or (ii) they started first‐line therapy after 25 May2016. Data from NSCLC patients with uncommon *EGFR* mutations (including single or complex mutations) were collected by an independent site‐monitoring organization (EP‐SOGO Co., Ltd.) and were subsequently analyzed. Patient accrual ended on 31 October 2018 and the data cutoff time was 31 October 2019. The study protocol (UMIN000028989) was approved by the ethics committee of the participating institutions.

### Clinical assessments

The antitumor response to treatment was assessed on the basis of the Response Evaluation Criteria in Solid Tumors (version 1.1) using computed tomography (CT). For cases without CT examination but wherein clinical symptoms or chest X‐ray suggested PD, PD onset was defined as the date when the attending physician clinically evaluated PD. Progression‐free survival (PFS) was defined as the interval from the start of any systemic therapy to patient death or PD detection. Overall survival (OS) was defined as the period from the start of any systemic therapy to the date when the patient died or was last known to be alive. Duration of treatment (DOT) was defined as the interval from the start of each treatment to the date when the treatment was discontinued or was last known to be given.

### Statistical analysis

PFS, OS, and DOT curves were estimated using the Kaplan–Meier method. The log‐rank test was used to compare survival between groups. Statistical analyses were performed using SPSS software version 22.0 (IBM, Chicago, IL, USA).

## Results

### Patient characteristics

A total of 23 of the 542 patients whose data were collected had uncommon *EGFR* mutations. Of them, 22 received EGFR‐TKIs between May 2016 and October 2019. Patient demographics are summarized in Table 1. All patients were diagnosed with adenocarcinoma. The median age was 72.5 (range, 54–86) years. A total of 13 patients (56.5%) were female, and 10 patients (43.5%) were never‐smokers; 18 patients were treated with EGFR‐TKIs as first‐line therapy, and four patients received EGFR‐TKIs as second‐ or late‐line therapy. A total of 10 patients were treated with first‐generation (gefitinib = 6, erlotinib = 4) and 12 were treated with second‐generation (afatinib) EGFR‐TKIs.

**Table 1:** Patient demographics

		*N* = 23	%
Age, years old	Median (range)	73 (54–86)	
Gender	Male / female	10 / 13	43.5 / 56.5
Smoking status	Never / ever	10 / 13	43.5 / 56.5
Stage	III / IV • recurrence	4 / 19	17.4 / 82.6
Histology	Adenocarcinoma / others	23 / 0	100 / 0
*EGFR* mutation	Exon 20 insertion	2	8.7
	Single point mutation	17	73.9
	G719X (G719A / G719S / G719C)	13 (8/1/4)	
	L861Q	4	
	Compound mutation	4	17.4
ECOG performance status	0–1 / ≥2	22 / 1	95.7 / 4.3
Line of first EGFR‐TKI	Not used / first / ≥second	1 / 18 / 4	4.3 / 78.3 / 17.4
Regimen of first EGFR‐TKI^†^	Gefitinib / erlotinib / afatinib	6 / 4 /12	27.3 / 18.2 / 54.5

^†^ *N* = 22.

### Response per EGFR‐TKI generation

There were 10 patients who received first‐generation EGFR‐TKI (gefitinib or erlotinib) therapy (first‐line = 8, second‐ or late‐line = 2). The response to this therapy was favorable (RR = 60%, DCR = 90%). In contrast, 12 patients received second‐generation EGFR‐TKI (afatinib) therapy (first‐line = 10, second‐ or late‐line = 2), with likewise favorable response (RR = 58.3%, DCR = 83.3%).

**Figure 1:**
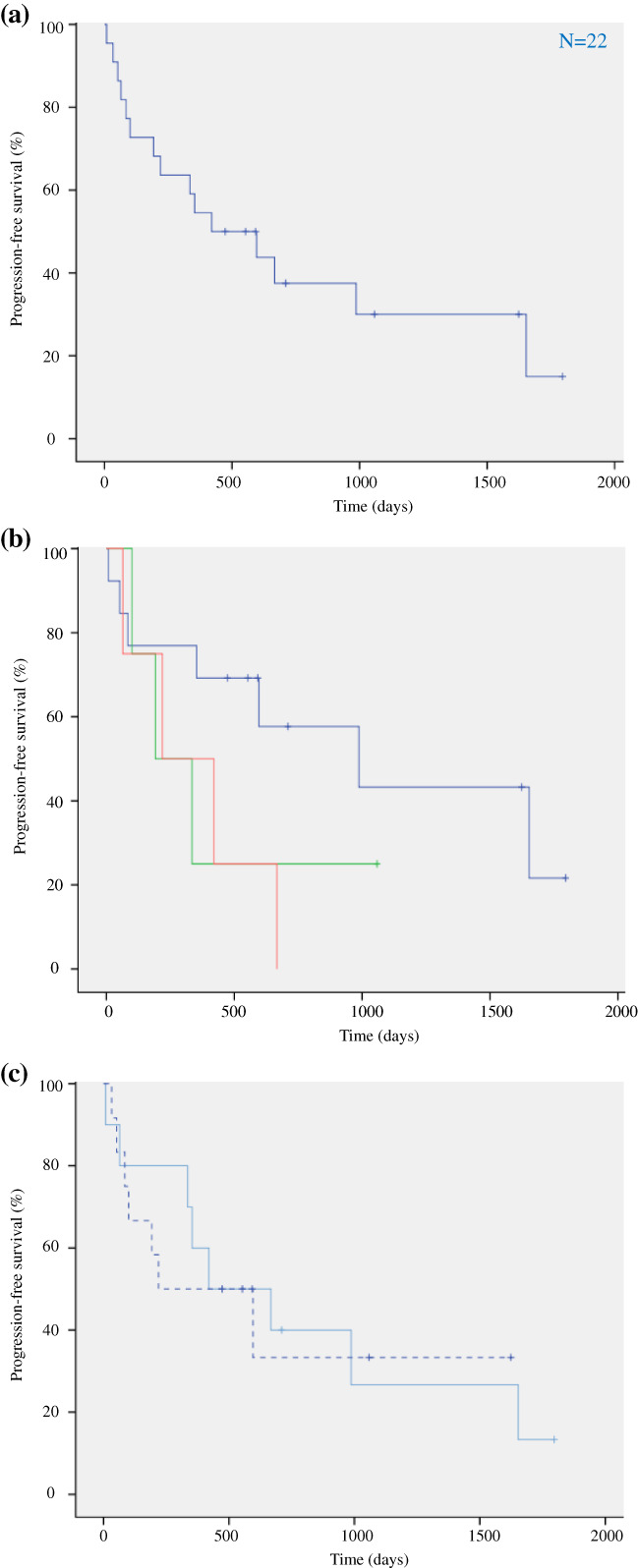
Kaplan–Meier curves for progression‐free survival (PFS) following first‐line epidermal growth factor receptor‐tyrosine kinase inhibitor (EGFR‐TKI) therapy in patients with uncommon *EGFR* mutations. (**a**) PFS of all patients with uncommon *EGFR* mutations (*N* = 22). (**b**) PFS of patients with G719X, L861Q, and compound mutations [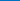 G719X (*N* = 13), 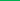 L861Q (*N* = 4), 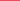 Compound mutation (*N* = 4)]. (**c**) PFS of patients harboring uncommon *EGFR* mutations treated with first‐ and second‐generation EGFR‐TKIs [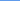 First‐generation EGFR‐TKI (*N* = 10), 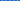 Second‐generation EGFR‐TKI (*N* = 12)].

**Figure 2:**
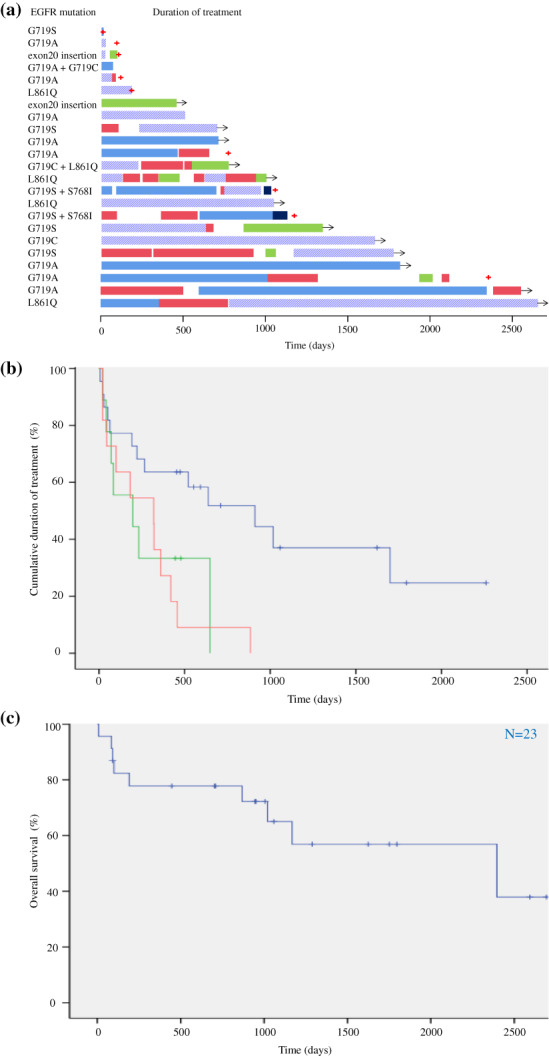
The entire clinical course and overall survival (OS) in patients with uncommon mutations. (**a**) The treatment durations of epidermal growth factor receptor‐tyrosine kinase inhibitor (EGFR‐TKI), immune checkpoint inhibitor (ICI), and cytotoxic chemotherapy (CCT) were indicated in each patient harboring uncommon *EGFR* mutations [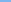 First‐generation EGFR‐TKI, 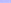 Second‐generation EGFR‐TKI, 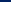 Third‐generation EGFR‐TKI, 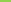 ICI, 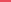 CCT, 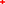 Death, 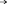 Ongoing treatment]. (**b**) Kaplan–Meier curves for cumulative duration of treatment 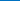 EGFR‐TKI (*N* = 22), 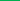 ICI (*N* = 9), 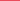 CCT (*N* = 11); and OS (**c**) in patients with uncommon *EGFR* mutations (*N* = 23).

There was no significant difference in PFS between the first‐generation (*n* = 10, median PFS = 14.0 months [95% CI: 0–30.2]) and second‐generation (*n* = 12, median PFS = 7.3 months [95% CI: 0–22.5]) EGFR‐TKIs (Fig 1c). Nevertheless, numerous patients have continued receiving EGFR‐TKI therapies, especially those in the afatinib cohort (Fig 2a); hence, the data presented may be premature.

### Response to EGFR‐TKIs per mutation subcategory

The overall response rate (RR) and disease control rate (DCR) of NSCLC patients with uncommon *EGFR* mutations who received first EGFR‐TKI therapy was 59.1% and 81.8%, respectively (Table 2). A total of 13 patients exhibited partial response (PR). Within the single mutation subcategory, nine out of 13 patients with G719X mutation (RR = 69.2%, DCR = 84.6%) and two out of four patients with L861Q mutation (RR = 50.0%, DCR = 100%) exhibited PR. In contrast, within the compound mutation subcategory, two out of four patients exhibited PR (RR = 50.0%, DCR = 75.0%).

**Table 2:** Response to EGFR‐TKIs per mutation subcategory

	Exon 20 insertion (*N* = 1)	G719X (*N* = 13)	L861Q (*N* = 4)	Compound mutation (*N* = 4)	All (*N* = 22)
CR	0	0	0	0	0
PR	0	9	2	2	13
SD	0	2	2	1	5
PD	1	0	0	1	2
NE	0	2	0	0	2
RR (%)	0	69.2	50.0	50.0	59.1
DCR (%)	0	84.6	100.0	75.0	81.8

CR, complete response; DCR, disease control rate; NE, not evaluated; PD, progressive disease; PR, partial response; RR, response rate; SD, stable disease.

The median PFS following first EGFR‐TKI therapy was 14.0 months (95% confidence interval [CI]: 3.2–25.8) in all patients with uncommon mutations (Fig 1a). No significant difference in PFS was observed between the single and compound mutation subcategories. Nevertheless, the PFS of patients with G719X mutation was favorable (*n* = 13, median PFS = 32.9 months [95% CI: 3.1–62.6]), compared to those with L861Q (*n* = 4, median PFS = 6.4 months [95% CI: 0–14.1]) or compound (*n* = 4, median PFS = 7.3 months [95% CI: 0–18.9]) mutations (Fig 1b).

### Cumulative DOT and OS in patients with uncommon mutations

The median cumulative DOT for EGFR‐TKI therapy was 30.4 months (*n* = 22, 95% CI: 8.6–52.1). This was longer than that of CCT (*n* = 11, median DOT = 10.7 months [95% CI: 2.7–18.6]) or ICI (*n* = 9, median DOT = 6.6 months [95% CI: 0–17.6]) (Fig 2a,b). Furthermore, the median OS from the start of any systemic treatment (except for pre‐ and postoperative chemotherapy or chemoradiotherapy) was 79.9 months (95% CI: 5.4–154.3) (Fig 2c). Nevertheless, numerous patients have continued to receive therapies; hence, the data presented may be premature.

## Discussion

This study demonstrated that both first‐ and second‐generation EGFR‐TKIs elicit clinical responses in NSCLC patients with uncommon mutations. According to previous reports, first‐generation EGFR‐TKIs were less effective in NSCLC patients with uncommon mutations than in those with common mutations.^12^, ^13^, ^14^, ^15^ Furthermore, Chiu *et al*. reported that first‐generation EGFR‐TKI treatment efficacy was similar among patients with uncommon single mutations (G719X: median PFS = 6.3 months, L861Q: median PFS = 8.1 months).^14^ In contrast, afatinib—an irreversible second‐generation EGFR‐TKI—elicited better responses in patients with G719X mutation than in those with L861Q mutation.^16^, ^17^, ^18^ Our results are consistent with those of previous reports in that EGFR‐TKIs elicited only moderate activity in patients with L861Q mutation. By contrast, EGFR‐TKIs appear to have elicited favorable activity in patients with G719X mutation (Fig 2a,b), but this data may be premature because numerous patients have continued receiving EGFR‐TKI therapy, especially in the afatinib cohort. Afatinib is currently in phase III testing to investigate whether this second‐generation EGFR‐TKI can improve clinical outcomes for NSCLC patients with uncommon mutations compared with platinum doublet chemotherapy.^19^ Moreover, a recent report indicated that osimertinib, a third‐generation EGFR‐TKI, also elicited favorable activity in NSCLC patients with uncommon mutations.^20^ In our study, only two patients were treated with osimertinib as late‐line therapy (Fig 2a); thus, its effect in this cohort was unclear.

The clinical effectiveness of CCT or ICI in patients with uncommon *EGFR* mutations also remains unclear. We therefore surveyed all clinical courses as well as OS in this cohort. Watanabe *et al*. reported that in a cohort of patients receiving carboplatin plus paclitaxel therapy, the OS of patients with uncommon *EGFR* mutations was similar to those of patients with common mutations; in another patient cohort (receiving gefitinib therapy) the OS of those with uncommon mutations was shorter than those with common mutations.^13^ Moreover, several reports showed that ICIs conferred less sensitivity in *EGFR*‐mutated NSCLC.^21^, ^22^ However, patients with uncommon *EGFR* mutations had better response and long‐term survival.^23^, ^24^, ^25^ Yamada *et al*. showed that patients with uncommon *EGFR* mutations had significantly better PFS (median PFS = 8.5 months) compared to those with common mutations (median PFS = 1.7 months).^25^ In our study, ICIs elicited moderate responses in patients with uncommon EGFR mutations. Moreover, several patients did not exhibit PD during ICI therapy. Our results indicate that in addition to EGFR‐TKIs, CCT and ICIs contribute to long‐term disease control in patients with uncommon mutations.

This study has several limitations. First, the sample size was small because of the rare nature of the mutation being studied. Previous studies also had sample sizes similar to our own. Moreover, despite this limitation, we believe that our results are relevant because we focused on specific patient characteristics, mutation status, and the entire clinical course. Second, our study was retrospective and only included Japanese patients. There may be various patient condition biases when each treatment was initiated.

In summary, both first‐ and second‐generation EGFR‐TKIs elicit favorable responses in NSCLC patients with uncommon *EGFR* mutations. Further studies are warranted to develop the optimal treatment strategy in this rare cohort.

## Disclosures

This research was supported by AstraZeneca. M Kanazu reports speaker's fees from AstraZeneca and Chugai. M Mori reports speaker's fees from AstraZeneca, Chugai, and Boehringer Ingelheim. K Nishino reports personal fees from AstraZeneca, Chugai, Pfizer, and Boehringer Ingelheim and grants from Boehringer Ingelheim. I Nagatomo reports personal fees and grants from AstraZeneca and Chugai. H Suzuki reports personal fees from AstraZeneca and Chugai. T Hirashima reports speaker's fees from AstraZeneca, Chugai, and Pfizer and research funds from AstraZeneca and Chugai. T Kumagai reports speaker's fees and research funds from AstraZeneca, Chugai, Pfizer, and Boehringer Ingelheim. F Imamura reports honoraria from AstraZeneca, Chugai, Pfizer, and Boehringer Ingelheim and research funds from AstraZeneca. M Kimura, T Shiroyama, S Ihara and K Komuta report no conflict of interest. The authors have no other relevant affiliations or financial involvement with any organization or entity with a financial interest in or financial conflict with the subject matter or materials discussed in the manuscript apart from those disclosed.
